# Assessment of Oxidative Stress and Biometric Data in a Captive Colony of Hamadryas Baboons (*Papio hamadryas* Linnaeus, 1758) at the Ravenna Zoo Safari (Italy)

**DOI:** 10.3390/vetsci12050466

**Published:** 2025-05-13

**Authors:** Barbara Biancani, Monica Carosi, Michele Capasso, Giacomo Rossi, Simona Tafuri, Francesca Ciani, Chiara Cotignoli, Francesco Zinno, Elena Venturelli, Matteo Galliani, Federica Spani

**Affiliations:** 1 Department of Veterinary Medicine and Animal Production, University of Naples Federico II, 80138 Napoli, Italy; capassovet@gmail.com (M.C.); simona.tafuri@unina.it (S.T.); ciani@unina.it (F.C.); franz.zinno@gmail.com (F.Z.); 2 School of Biosciences and Veterinary Medicine, University of Camerino, 62024 Matelica, Italy; giacomo.rossi@unicam.it; 3 Department of Science, Roma Tre University, 00146 Rome, Italy; monica.carosi@uniroma3.it; 4 Zoo Safari Ravenna, 48125 Ravenna, Italy; ccotignoli@gmail.com (C.C.); venturellielena017@gmail.com (E.V.); matteogalliani11@gmail.com (M.G.); 5 Department of Science and Technology for Sustainable Development and One Health, Università Campus Bio-Medico di Roma, 00128 Rome, Italy; f.spani@unicampus.it

**Keywords:** reactive oxygen species (ROS), physiological biomarkers, reproductive metabolism, health monitoring, primate aging, welfare assessment

## Abstract

This study evaluates the health of a captive colony of Hamadryas baboons at Ravenna Zoo Safari (Italy), focusing on oxidative stress markers and biometric data. Forty-eight individuals were assessed during routine veterinary procedures: males underwent vasectomy, and females were checked for pregnancy. Biometric data collected included body weight, body length, and genital measurements in males, while females were evaluated for reproductive status. Oxidative stress was measured using two tests that assess both harmful pro-oxidant levels and the body’s antioxidant defenses. Results showed no significant differences in oxidative stress levels between sexes, although males and females differed in body weight. Pregnant and postpartum females exhibited higher oxidative stress, likely due to the metabolic and hormonal demands of reproduction. This supports the idea that reproductive activity increases the production of reactive oxygen species, requiring stronger antioxidant responses. In males, correlations between body weight and genital measurements suggest these could help estimate age in the absence of birth records. No link was found between oxidative stress and body weight, indicating limited age-related effects on these markers. Overall, the study highlights the importance of monitoring oxidative stress in captive primates to better understand the effects of reproduction and aging, and to improve welfare and management practices.

## Simple Summary

This study examined the health of a group of Hamadryas baboons living in captivity at Ravenna Zoo Safari in Italy. Researchers looked at the animals’ body measurements and levels of oxidative stress—an imbalance in the body between harmful molecules and the systems that defend against them. Forty-eight baboons were checked during routine health procedures. Males were sterilized, and females were checked for pregnancy. Scientists found that, although male and female baboons had different body weights (used as a proxy to classify individuals of unknown age), their levels of oxidative stress were similar. However, pregnant and recently pregnant females showed higher stress levels, likely because of physical changes related to reproduction. In males, certain body measurements were found to be helpful in estimating age, which is useful when birth records are missing. The study found no link between body weight and oxidative stress, suggesting that aging may not strongly affect these markers. These results show the value of monitoring oxidative stress to improve the care of baboons in captivity and better understand how reproduction and aging affect their health. Future research may help develop simple, non-invasive ways to assess animal health and well-being in zoos.

## 1. Introduction

Hamadryas baboon (*Papio hamadryas* Linnaeus, 1758) is a primate species belonging to the Cercopithecidae family, listed as “Least Concern” on the IUCN Red List. While their populations are currently stable, they face threats from habitat loss and human conflict, especially in agricultural areas where they are considered pests. Conservation efforts focus on mitigating these conflicts and preserving their natural habitats [1]. This species lives in the southern peninsula of Arabia and north-eastern Africa—primarily Ethiopia—inhabiting diverse environments including arid regions, savannas, and rocky areas near water sources. Known for their unique social structure, they live in a multi-level society characterized by multiple nested social levels, starting from the smaller One Male Units (OMUs) dominated by a single male, which aggregate into progressively larger clans, bands, up to troops of about 200 individuals [2,3] in a hierarchical social system that helps them manage resources and defend against predators [4]. Hamadryas baboons exhibit a variety of social behaviors, including grooming to establish bonds, threat displays like staring and yawning to show canines, and vocalizations for communication [4]. Hamadryas live in a male-dominated, highly competitive society, characterized by intense male–male competition in a polygynous reproductive system, and small OMUs are managed through aggressive coercion directed at females. In this highly competitive regime, hamadryas baboons are sexually dimorphic as for body weight, canine size, and mane occurrence [5]. Their diet is omnivorous, primarily consisting of plant matter such as fruits, grasses, roots, seeds, and occasionally small mammals and insects [6].

Because of their close genetic relationship [7,8] to humans and similar physiological traits, various baboon species are frequently used as model organisms in biomedical research. These species, until promising alternatives are validated, are still good non-human primate models which may greatly help in studies addressing cardiovascular diseases, infectious diseases such as HIV/AIDS, tuberculosis, and hepatitis, as well as neurological disorders [9,10,11,12,13]. Their immune system’s resemblance to that of humans allows researchers to explore disease progression effectively. Additionally, their social behaviors and complex brain structures offer significant insights into human psychiatric and neurological conditions [14,15,16]. Investigating oxidative stress in *P. hamadryas* is crucial, as it plays a significant role in the pathogenesis of most of these conditions. Understanding the biological significance of oxidative stress may help in developing strategies for the prevention and treatment of oxidative stress-related diseases in humans through lifestyle changes, dietary supplements, and pharmacological interventions that enhance antioxidant defenses [17].

Oxidative stress occurs when there is an imbalance between the production of reactive oxygen species (ROS) and the body’s ability to neutralize these reactive intermediates or repair the resulting damage. This imbalance can severely affect biological systems, as ROS can damage lipids, proteins, and DNA, leading to cellular dysfunction, apoptosis, or necrosis. This damage contributes to several diseases and the aging process [18]. As oxidative stress accumulates over time, it impairs cellular functions, promoting aging and the onset of chronic diseases such as diabetes, cardiovascular diseases, and atherosclerosis [19]. Additionally, oxidative stress is linked to neurodegenerative disorders like Alzheimer’s and Parkinson’s disease, where oxidative damage to neurons plays a crucial role in disease progression [20]. While ROS can be harmful, they also play a crucial role in the immune response. Phagocytic cells, such as polymorphonuclear cells and macrophages, produce ROS to eliminate pathogens. Additionally, oxidative stress is involved in activating inflammatory pathways by increasing ROS production, creating a feedback loop that contributes to chronic inflammation and related diseases [21]. Under normal conditions, the body has antioxidant defense mechanisms, including enzymatic antioxidants like superoxide dismutase (SOD), catalase (CAT), and glutathione peroxidase (GPx), as well as non-enzymatic antioxidants such as vitamin C and E, and glutathione. These antioxidants neutralize ROS, helping to mitigate oxidative stress [22].

The aim of the present study was to assess the health status of a captive hamadryas baboon housed at the Safari Ravenna Zoo (Italy) by means of oxidative stress biomarkers, thereby contributing data to an underrated indicator of welfare as well [23]. Specifically, we were interested in evaluating oxidative stress levels in the animal subjects in relation to sexual dimorphism (i.e., body weight and reproductive state) and aging, also useful for management purposes. Previous studies have highlighted discrepancies between field and captive studies on baboons. Captive environments (e.g., controlled diets and a closed social environment can introduce biases, especially in behavioral studies) [24] may not accurately reflect the growth patterns, age classifications [25], and oxidative stress conditions experienced by wild baboons. Differences in diet and growth rates can impact the balance between prooxidants and antioxidants, thereby influencing the oxidative status of individuals [26,27]. For this purpose, and to compensate for the lack of exact birthdate information, a variety of biometric data were used to assist in supporting growth data consistency, originally based on body weight.

## 2. Materials and Methods

### 2.1. Animals, Housing, and Management

The social group of hamadryas baboons in Safari Ravenna Zoo (Savio di Ravenna, Italy) counted about N = 54 individuals, housed in an indoor recovery and outdoor exhibit. Captive diet consisted of fresh fruit and vegetables daily, alfalfa hay daily, and boiled chicken and rice once a week.

The founders of this group were moved into Safari Ravenna Zoo from another zoo in 2008, and breeding was freely allowed over the years. No external animals were added to the group since its arrival; thus, inbreeding had been progressing thereafter. Due to the increase in the number of animals in the group and the inbreeding situation, the Safari Ravenna finally decided to apply a permanent reproduction control by sterilizing males, also preventing a reduction of welfare conditions caused by an increasing animal density in the exhibit. Microchipping of all the individuals (both males and females) was also performed.

### 2.2. Data Collection

This study took advantage of a scheduled vasectomy procedure involving males from a highly inbred captive group of hamadryas baboons. Although the anesthesia protocol may be designed to minimize distress, anesthesia still represents a stressful experience for captive primates [28]. Therefore, it is considered good practice to use such occasions to conduct concurrent medical examinations and collect as much data as possible for additional purposes (e.g., animal management, welfare, and scientific research), thereby reducing the need for further captures. For this reason, females have also been included in the sample and some biometric data were collected as part of a concurrent study on external genital characteristic adaptive function.

Between December 2023 and January 2024, forty-eight hamadryas baboons (N males = 28; N females = 20) were anesthetized and taken into veterinary clinic for microchipping procedure, medical checkups, and finally, vasectomy. Three infants were still dependent on their mothers at the time of data collection, and, therefore, both the infants and their mothers (*n* = 6) were not included in this study for ethical and welfare reasons. The whole procedure was deemed necessary for management, medical, and welfare reasons. In accordance with Guideline 1999/22/EG of the European Union, zoological gardens are scientific institutions and obtaining ethical approval was not necessary [29]. For all the hamadryas baboons, anesthetics were administered remotely using inject Blowpipes hingperformace B16 (TELEDART GMBH & Co. KG, Westheim, Germany). Three anesthetic protocols were used dividing the animals in three groups: tiletamine-zolazepam (3 mg/kg) was used in combination with dexmedetomidine (0.02 mg/kg); dexmedetomidine (0.03 mg/kg) was associated to ketamine (6 mg/kg) and methadone (0.2 mg/kg); dexmedetomidine (0.05 mg/kg) was associated to midazolam (1 mg/kg) and methadone (0.2 mg/kg) [30,31]. Once in the veterinary clinic, an endovenous catheter was placed into saphenous or cephalic vein and, when necessary, maintenance was achieved using propofol total intravenous anesthesia (TIVA) on intubated patients. Blood samples for the oxidative stress study were collected immediately after anesthesia from femoral artery in all individuals with 21-gauge butterfly needles for CBC and chemistry panel. Blood was then centrifuged (2500 rpm for 15 min) to separate the serum, and serum aliquots not used for chemistry panel were kept frozen at −20 °C until analyses were performed within two months from collection.

Several biometric parameters were measured in males (body length, testicular volume, penile and baculum length), who then underwent X-ray imaging of their external genitalia followed by vasectomy. In contrast, females were only weighed and subjected to ultrasonographic examination to determine potential pregnancy status. The disparity in data collection and analyses between males and females was due to differences in the invasiveness of the procedures. Specifically, the males required vasectomy, which necessitated longer anesthesia duration. This extended time allowed for the collection of a broader range of biometric data. Conversely, females only underwent microchipping and abdominal ultrasonography, limiting the available time for additional data collection compared to the males.

### 2.3. Biometric Data in Males

Males were weighed, and measurements of body length, testis volume, and penis length were recorded. Body length was measured using a measuring tape from the top of the skull (parietal bone) to the base of the tail (sacrum). Testis volume was calculated using the formula as follows [32]:

$$10^{-3}\times {\left(\frac{testicular\;breadth}{2}\right)}^{2}\times \left(\frac{testicular\;length}{2}\right)\times \frac{4\pi }{3}$$

**Figure 1:**
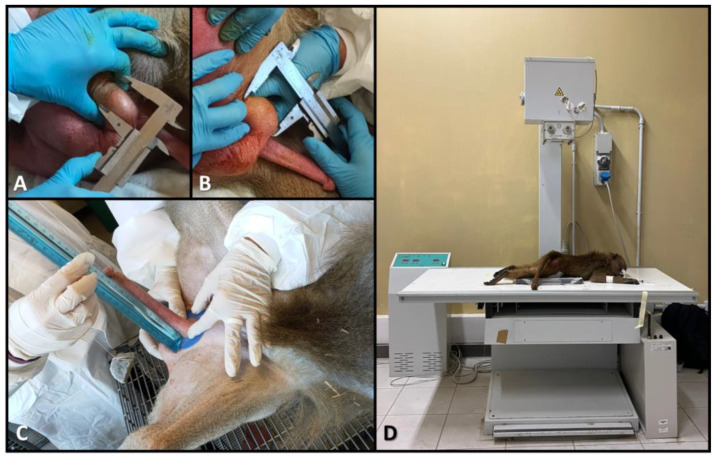
(**A**,**B**): Measurement of testicular breadth (**A**) and length (**B**) in *Papio hamadryas*. (**C**) Measurement of penis length using the method described by Carosi et al. [33]. (**D**) Positioning of a *P. hamadryas* individual on the scanning table for X-ray acquisition of the penile bone (baculum).

Testicular breadth and length were measured using a manual caliper (Figure 1A,B). Penis length was determined following the method described by Carosi et al. [33], as illustrated in Figure 1C. Briefly, a plastic washer was placed at the base of the penis, ensuring it adhered closely to the pubis. A thin string was looped just below the glans, and a small weight (48 g) was attached to the other end of the string to apply traction to the penis. To maximize elongation, the string with the attached weight was passed over a plastic tube, simulating a pulley system. The total length was then measured using a rigid ruler placed perpendicular to the plastic washer and kept parallel to the string to standardize the measurements.

For measuring the length of the penile bone (baculum), males underwent Radiographic examination using the Somedic X-ray unit (Somaglia (LO), Italy) with the following scan parameters: tension = 55 kV; current = 6.0 mAs. Each individual was positioned prone on the scanning table with legs spread apart (Figure 1D) to allow the external genitalia to extend and fully adhere to the surface of the table. This positioning prevented interference from other skeletal structures in the imaging field and ensured consistent positioning of the individuals (and thus their external genitalia), allowing for comparable measurements.

**Figure 2:**
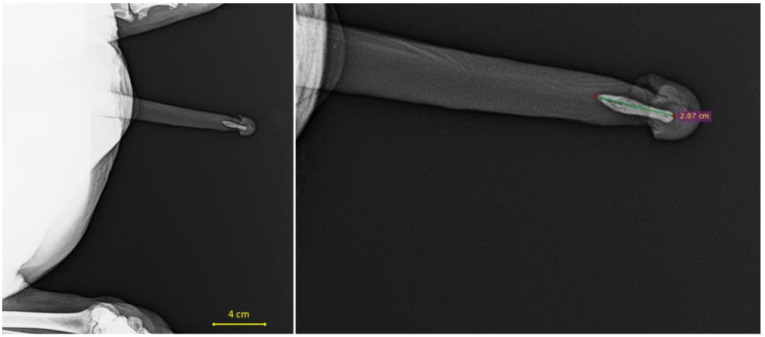
X-ray scans showing the baculum (penis bone) of a *Papio hamadryas* individual. The (**left**) image provides a general view of the basin, while the (**right**) image displays the measurement of the baculum length (2.07 cm), as indicated by the green line.

Subsequently, the tomographic scans were processed using RadiAnt DICOM Viewer 2024.1 software, and data on the baculum length for each scanned individual were obtained by measuring the distance between two points placed at the proximal and distal ends of the penile bone (see Figure 2).

All biometric data were collected in triplicate by two independent operators (FS, MC), and the averages of these measurements were used for subsequent statistical analyses.

### 2.4. Oxidative Stress Data in Males and Females

Several markers are used in the literature to measure oxidative status across different species [23].

The measurement of oxidative stress was evaluated using the d-ROMs test, which determines the pro-oxidative status by measuring hydroperoxides in the serum, and the OXY Adsorbent test that measures the antioxidant barrier in serum samples. The d-ROMs test determines the level of oxidative stress by measuring the amount of organic hydroperoxide (ROOH), a subclass of reactive oxygen metabolites (ROM). The test measures the oxidant ability of a serum sample using a chromogen as an indicator (N,N-diethyl-p-phenylenediamine), producing a pink-colored derivative that is photometrically quantified at 505 nm [34,35]. The intensity of the developed color is directly proportional to the concentration of ROMs, according to Lambert–Beer’s law, and is expressed as Carratelli Units (1 CARR U = 0.08 mg hydrogen peroxide/dL).

The antioxidant barrier was measured using the OXY Adsorbent test, which measures the ability of the sample to counteract the oxidation induced by a solution of hypochlorous acid (HClO). Unreactive HClO radicals further react with the chromogen solution of N,N-diethyl-p-phenylenediamine and produce a colored complex, which is measured at 505 nm. The results were expressed as μmol HClO/L [36,37]. The oxidative stress kits were purchased from Diacron International, Grosseto (Italy).

Oxidative Stress index (OSi) is an arbitrary value given by the ratio between the values of d-ROMs and OXY (×100), used as an indicator of oxidative stress degree [35].

### 2.5. Statistical Analysis

A comprehensive descriptive statistical analysis was conducted to summarize all collected variables for both sexes. For each variable, measures of central tendency (mean and median) and dispersion (standard deviation, minimum, and maximum) were calculated. These analyses provided an initial overview of the dataset and facilitated the identification of potential outliers.

The normality of each variable within the male and female groups was assessed using the Shapiro–Wilk test [38]. This test was employed to determine the appropriate statistical methods for group comparisons, as parametric tests require normally distributed data. In cases whose data were not normally distributed, the Z-score normalization method, also known as standardization, was applied. This is a statistical method used to rescale data so that they have a mean of 0 and a standard deviation of 1. This transformation is particularly useful for comparing data points across variables that have different units or scales, as in the present study.

To examine relationships among morphological variables in males and in order to measure their consistency during growth and mature life, a Pearson correlation analysis was performed [39] on standardized data (*p* < 0.05). Morphological measurements, including body weight, body length, testis volume, penile length, and baculum length, were included in the analysis.

Differences between males and females were assessed for body weight and oxidative stress biomarkers. A non-parametric Mann–Whitney U test [40] was used to assess differences in means calculated on raw data. The analysis included all oxidative stress parameters such as d-ROMs, OXY, and OSi, alongside body weight measurements.

To evaluate the oxidative stress status of the group relative to human health standards [41,42,43], the mean values of oxidative stress biomarkers for the entire group were compared with reference values derived from the scientific literature. Human reference values were used because no specific reference ranges currently exist for baboons in managed care, and this species is frequently employed as a model in studies of human pathologies.

A paired Wilcoxon signed-rank test [44] was used for each variable to determine whether the group’s mean values significantly deviated from these thresholds. This non-parametric test was chosen due to the small sample size and the potential non-normality of the data. The results provided insights into the health status of the colony compared to established human benchmarks.

To model oxidative stress in relation to sexual dimorphism and reproductive state, a Spearman rank correlation analysis was conducted using body weight as a proxy for growth (and more generally, for age), the only available morphometric variable common to both sexes. This analysis examined the relationship between oxidative stress parameters (d-ROMs, OXY, OSi) and body weight. Spearman’s method was chosen due to its robustness against non-linear relationships and non-normal distributions. The analysis aimed to explore whether body weight, as an indirect indicator of growth (and to some extent of age), influences oxidative stress biomarkers in the colony. All statistical analyses were conducted using R statistical software (version 4.5.0).

## 3. Results

Abdominal ultrasonography performed on the females identified two pregnant individuals and one who had recently given birth. (EV pers. obs.).

**Figure 3:**
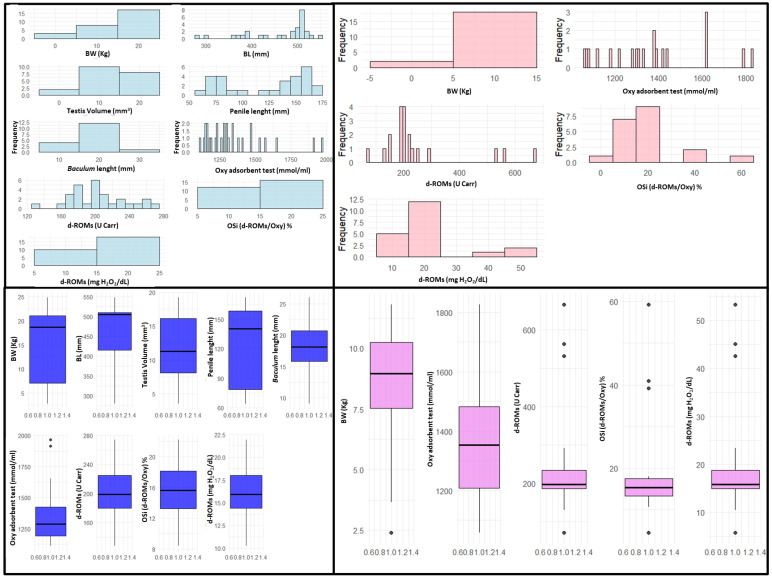
Frequency histograms (**top**) and box plots (**bottom**) of biometric and oxidative stress data collected from males (light and dark blue, (**left**)) and females (light and dark pink, (**right**)). BW = body weight; BL = body length; OXY Adsorbent test = measurement of the antioxidant barrier in serum samples; d-ROMs (U Carr) = measurement of ROM expressed as Carratelli Units; OSi = Oxidative Stress index. The Shapiro–Wilk test, applied to assess whether the collected data followed a normal distribution, revealed that the distribution of some variables significantly deviated from normality (see Tables S1 and S2). In light of this, the data were standardized using the Z-score normalization method (see above).

The data collected from biometric measurements and oxidative stress analyses were summarized and described in Table 1. Visual representations, including histograms and boxplots, were created to analyze the distribution and variability of the data (Figure 3).

**Figure 4:**
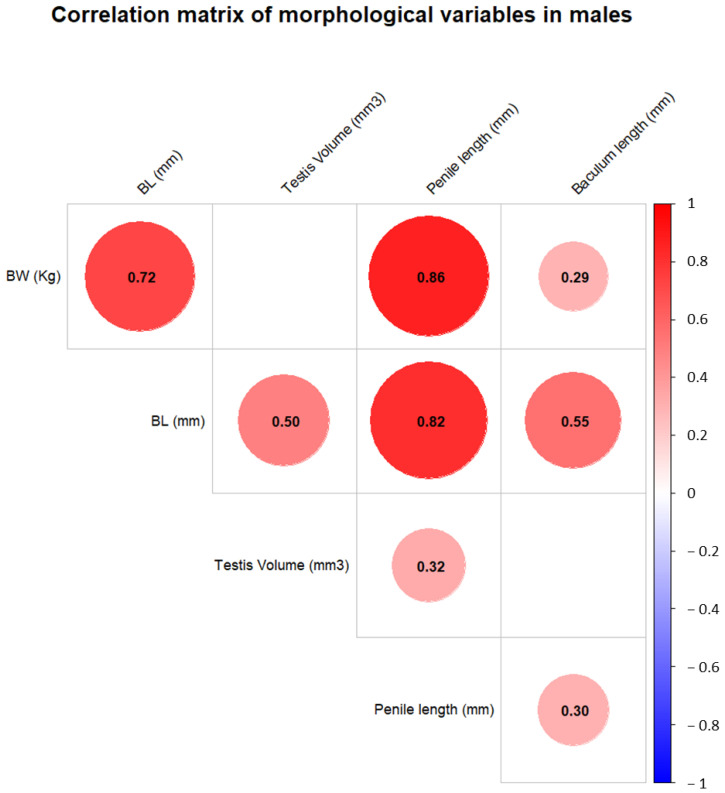
Correlation matrix of biometric variables in males. The matrix depicts Pearson correlation coefficients among body weight (BW), body length (BL), testis volume, penile length, and baculum length. Positive correlations are shown in red and negative correlations in blue, with the intensity reflecting the strength of the correlation. Only significant correlations (*p* < 0.05) are visualized.

Following the Pearson correlation analysis performed on the biometric data collected from the males, a correlation matrix was generated to quantify the strength and direction of associations between these variables. Statistical significance was set to <0.05 and assessed for each correlation coefficient (see Table S3). Results were visualized using a heatmap (see Figure 4) to highlight significant relationships. The results show that almost all variables are correlated with each other, except for testis volume, which was not correlated with either body weight or baculum length.

The Mann–Whitney U test, performed on the variables common to both males and females, revealed a single significant difference between the sexes in body weight (sexual dimorphism). All other oxidative stress variables analyzed showed no significant differences in values between males and females (Table 2).

Since no sexual dimorphism was found in the oxidative stress values, subsequent analyses were conducted by combining all the colony data into a single dataset, no longer considering sex as a factor. To statistically assess differences, the paired Wilcoxon signed-rank test was performed between the mean values of each oxidative stress biomarker and a single reference value, chosen as the upper limit of the normal range reported in the literature. Although all mean values were significantly different from the selected reference points, they remained within the corresponding normal ranges (Table 3).

This comparison between the observed values and the reference values was also performed for each individual to detect any specific cases falling outside the normal range. Three female individuals showed d-ROM values, both in terms of U Carr and H_2_O_2_/dL, significantly above the normal threshold for humans (see Table S4). These females were the same individuals identified as pregnant (N = 2) through ultrasonographic examination, along with one that had recently given birth. Finally, the Spearman rank correlation analysis performed between body weight and the oxidative stress variables showed no significant correlation, indicating an absence of oxidative stress variability related to the individuals’ growth (at least in terms of weight, used here also as a proxy for age).

## 4. Discussion

The evaluation of oxidative stress levels in a captive colony of *Papio hamadryas* provides valuable insights into the physiological and metabolic adaptations of this animal model, particularly in relation to reproduction and aging, contributing to the understanding of the biological significance of oxidative stress. In addition, such studies are also essential for understanding the health dynamics of captive populations, offering potential implications for both conservation efforts and the management of individuals under human care.

The Pearson correlation analysis of biometric data collected from males revealed significant correlations between most variables, with the notable exception of testis volume, which showed no correlation with either body weight or baculum length. This analysis was a crucial step in addressing the absence of exact birthdate information for the colony’s individuals under study. Given this limitation, the objective was to verify whether body weight, the most common measure of growth, could serve as an overall generic proxy for individual age. Although only partially interchangeable (body weight and age are linked ontogenetically but less in adulthood), body weight and age are often used interchangeably when the required variable is difficult or impossible to sample, as, for example, in studies on vocalization in wild primates, in which age and sex serve as proxy of body size [45]. The results demonstrated strong correlations of male body weight with all other biometric variables (body length, penile length, and baculum length) and all variables among them. This finding indicates that external genital dimensions can serve as effective biometric proxies for age in male individuals. Importantly, body weight, as a variable common to both males and females, also exhibited robust correlations with other biometric measures in males. This made body weight a viable and practical choice for subsequent analyses involving both sexes. By using body weight as a shared and correlated variable, we were able to incorporate data from both males and females into a unified framework for further analysis. This approach not only addressed the challenge posed by the lack of precise age data but also provided a methodologically sound basis for exploring age-related trends across the colony. The lack of certain correlations involving testis volume (e.g., with body weight and baculum length) may be explained by the numerous factors that influence testis size beyond body size. These include the spermatogenic activity of testicular tissue, which is itself affected by reproductive seasonality and social dominance status [46].

The Mann–Whitney U test, performed on the variables common to both males and females, revealed that body weight differed significantly between sexes. This result highlights the presence of sexual dimorphism in body weight, which is consistent with well-documented patterns in primate species in which males typically exhibit larger body sizes due to sexual selection pressures [47,48]. Contrary to other physiological measures [49], the lack of sexual dimorphism in oxidative stress variables between sexes suggests that oxidative stress levels are regulated similarly in males and females, regardless of their differing metabolic demands or reproductive roles [50]. This finding underscores the importance of considering physiological mechanisms that may buffer against oxidative stress across sexes to support overall health and reproductive success. Since no sexual dimorphism was found in the oxidative stress values, the data from both sexes were combined for further analysis. The paired Wilcoxon signed-rank test showed that, while the mean oxidative stress values differed from the reference values, they still fell within the normal range for humans. This suggests that the individuals in the colony were not experiencing abnormal oxidative stress levels. Finally, the Spearman rank correlation analysis between body weight and oxidative stress variables revealed no significant correlation, suggesting that oxidative stress levels do not vary with the individuals’ growth or weight. This finding implies that, within the studied colony, oxidative stress is not strongly influenced by age or body size, at least as estimated by weight, challenging the expectation that oxidative stress would increase with age-related metabolic changes. However, the limits deriving from the use of body weight as a proxy of age should not be overlooked, and this conclusion needs further evaluation.

ROS, generated as metabolic by-products [51,52], include superoxide radicals, hydrogen peroxide, hydroxyl radicals, nitric oxide radicals (NO•), and singlet oxygen. NO• plays key physiological roles in modulating blood flow, neural activity [53], and non-specific host defense, synthesized via nitric oxide synthase (NOS) [54,55,56,57]. ROS can also be produced non-enzymatically, such as during mitochondrial respiration or exposure to ionizing radiation [54,57]. While ROS are critical for cellular signaling, immune response, and reproduction [58,59,60,61], their excess can damage lipids, proteins, and nucleic acids, contributing to diseases like cardiovascular disorders, diabetes, and cancer [62,63,64].

ROS are essential in reproduction, facilitating hormone production, oocyte maturation, ovulation, and sperm-oocyte fusion [58,59,60,65,66]. However, their activity must be regulated by antioxidant systems, including SOD, CAT, and GPx [67]. Mitochondria are the primary ROS source, especially during high-energy-demand phases [68,69]. In humans, ROS levels are linked to aging due to reduced antioxidant bioavailability and cumulative cellular damage [70,71,72]. In non-human primates, oxidative stress correlates with reproductive activity, especially in males during competition and in multiparous females during lactation [73,74].

In vivo studies show species-specific ROS patterns. For example, oxidative stress increases with age in rhesus macaques (*Macaca mulatta* (Zimmermann, 1780)), especially in high-ranking males during mating [23,75], while chimpanzees (*Pan troglodytes* Blumenbach, 1775) prioritize reproductive activity over age-related ROS increase [76]. In vitro studies reveal that fibroblasts from longer-lived primate species resist ROS-induced apoptosis better than those from shorter-lived species [77]. Baboon fibroblasts exhibit sex-dependent responses to ROS, with female-derived cells showing greater resilience to stress and aging effects [78].

Regarding reproductive activity, the paired Wilcoxon signed-rank test between the mean values of each oxidative stress biomarker and the reference values showed that our results were different from those reported in literature (using both the human reference values and those reported in other studies) [41,42,43], but, nevertheless, within the normal range. To evaluate differences also at the individual level, the comparison between the observed and reference values was also performed for each individual: three female individuals showed d-ROM values, both in terms of U Carr and H_2_O_2_/dL, significantly higher than the normal threshold. These females were the same individuals identified as pregnant (N = 2) via ultrasound examination, along with one who had recently given birth. As reported by Georgiev et al. [73], our data show that reproductive activity is a significant factor in increasing ROS levels in *Papio hamadryas*, even in a controlled environment. Specifically, gestation, delivery, and maternal care significantly impact ROS production in females. Pregnancy leads to an increase in ROS due to hormonal and energetic changes. High progesterone levels during pregnancy promote ROS production through processes like the conversion of cholesterol into pregnenolone in mitochondria [79], and the subsequent conversion of pregnenolone to progesterone in the endoplasmic reticulum, which also produces ROS [80]. The metabolic demands of the growing fetus contribute to ROS production, with superoxide anions from placental mitochondria playing a key role in oxidative stress in the placenta [81]. Pregnancy induces significant physiological changes, including increased oxygen consumption and changes in energy utilization across organs, particularly the feto-placental unit [82]. The placenta, as the main source of ROS, plays a key role in maintaining maternal homeostasis [83]. Early in pregnancy, the placenta’s hypoxic environment produces ROS, which are crucial for placental blood flow and fetal nourishment [84,85]. The systemic inflammatory response in late pregnancy further increases ROS production [83,86]. A marked increase in oxygen concentration in the placenta at the end of the first trimester results in a significant rise in ROS production, especially in the trophoblast syncytium. Parturition also impacts ROS production, as shown in both human and animal studies [87,88]. Additionally, breastfeeding has a variable effect on ROS levels. It increases ROS production during the initial stages of milk production and nursing, with levels decreasing towards normal by the later stages [89]. During breastfeeding, metabolic shifts occur, including improved glucose management and lipid metabolism changes that initially elevate ROS production. This altered state persists for several weeks post-lactation before returning to baseline, supporting the “reset hypothesis” that lactation resets metabolic processes and reduces metabolic disease risk [90].

## 5. Conclusions

Our study carried out in a group of *Papio hamadryas* shows that, while no significant difference in the levels of oxidative stress can be seen in males of various ages and sexual development, the highest levels of ROS are observed in pregnant females or in the postpartum period. The return of females, far from the reproductive phase, to normal ROS values is consistent, according to our hypothesis, with Stuebe and Rich-Edwards’ reset hypothesis [90] which implies that breastfeeding reverses many of the metabolic changes that occur during pregnancy, allowing females to return to a non-reproductive baseline, and improving their metabolic condition long after reproduction has ended. Reactive oxygen species (ROS), naturally produced during mitochondrial energy metabolism, play a dual role as signaling molecules and potential sources of cellular damage when oxidative balance is disrupted. Theories such as the free radical and disposable soma suggest that ROS production and resource allocation to reproduction can compromise longevity [91]. While reproduction is hypothesized to increase oxidative stress due to heightened energy demands, direct evidence remains inconclusive [92,93,94,95,96]. Future research should aim to further elucidate the mechanisms underlying the oxidative stress response during reproduction and postpartum, particularly in captive populations, to better understand the long-term health impacts and potential adaptive advantages of these metabolic shifts. Expanding the study to include very young individuals could provide valuable insights into the early-life factors influencing oxidative stress. Additionally, identifying more reliable biomarkers of age for both males and females would enhance our ability to assess whether oxidative stress levels vary with age and to detect potential sex-based differences in oxidative stress dynamics across different life stages. Biomarkers of oxidative stress find application in a variety of research questions, and although still little used and validated as welfare indicators [23], recent advances towards non-invasive measurements should prompt scientists to pursue this methodological novelty, feasible both in captive and wild animals [97].

## Figures and Tables

**Table 1:** Descriptive and summary statistics of the raw data collected in this study. For each variable, where available, the mean, standard deviation (SD), median, minimum (Min), and maximum (Max) values are reported. NA: data not available.

	Males					Females				
Variable	Mean	SD	Median	Min	Max	Mean	SD	Median	Min	Max
BW (Kg)	15.22	7.36	18.7	2.75	24.85	8.49	2.54	8.98	2.4	11.8
BL (mm)	462.14	72.75	505	280	548	NA	NA	NA	NA	NA
Testis volume (mm^3^)	11.78	4.94	11.25	3.53	19.22	-	-	-	-	-
Penile length (mm)	123.11	39.11	140	64	172	-	-	-	-	-
*Baculum* length (mm)	17.81	4.34	18.1	9.05	26	-	-	-	-	-
OXY Adsorbent test (μmol HClO/L)	1346.03	218.11	1288.15	1111.2	1966.3	1370.92	227.81	1353.1	1058.6	1825.5
d-ROMs (U Carr)	203.71	33.67	199	128	274	251.25	153.36	198	72	666
OSi (d-ROMs/OXY × 100)	15.52	3.51	15.6	8.4	22.2	18.95	12.78	15.4	4.5	59.2
d-ROMs (mg H_2_O_2_/dL)	16.3	2.69	15.92	10.24	21.92	20.1	12.27	15.84	5.76	53.28

**Table 2:** Summary table of the results from the Mann–Whitney U test performed on the variables collected from males (M) and females (F): body weight (BW), OXY Adsorbent test = measurements of the antioxidant barrier in serum samples; d-ROMs (U Carr) = measures the level of ROM expressed as Carratelli Units; OSi = Oxidative Stress index.

Variable	Test Used	*p*-Value	Mean (M)	Mean (F)
BW (Kg)	Mann–Whitney	**0.009**	15.22	8.49
OXY Adsorbent test (mmol/mL)	Mann–Whitney	0.638	1346.03	1370.92
d-ROMs (U Carr)	Mann–Whitney	0.738	203.71	251.25
OSi (d-ROMs/Oxy) %	Mann–Whitney	0.958	15.52	18.94
d-ROMs (mg H_2_O_2_/dL)	Mann–Whitney	0.738	16.3	20.1

**Table 3:** Summary table of the results from the paired Wilcoxon signed-rank test performed on the mean values of oxidative stress parameters collected from the entire *P. hamadryas* group: OXY Adsorbent test = measurements of the antioxidant barrier in serum samples; d-ROMs (U Carr) = measures the level of ROM expressed as Carratelli Units; OSi = Oxidative Stress index.

Variable	Reference Values (in *Homo sapiens*)	Observed Mean	*p*-Value	Test Type
OXY Adsorbent test (mmol/mL)	>350	1356.4	7.1 × 10^−15^	Paired Wilcoxon Test
d-ROMs (U Carr)	<300	223.5	4.7 × 10^−6^	Paired Wilcoxon Test
OSi (d-ROMs/Oxy) %	<85.71%	16.9	1.7 × 10^−9^	Paired Wilcoxon Test
d-ROMs (mg H_2_O_2_/dL)	<24	17.9	4.7 × 10^−6^	Paired Wilcoxon Test

## Data Availability

All data are available as Supplementary Materials.

## Supplementary Materials

The following supporting information can be downloaded at https://www.mdpi.com/article/10.3390/vetsci12050466/s1, Table S1: Normality Test (Shapiro–Wilk) Results for Biometric and Oxidative Stress Variables in Male Hamadryas Baboons; Table S2: Normality Test (Shapiro–Wilk) Results for Biometric and Oxidative Stress Variables in Female Hamadryas Baboons; Table S3: Pearson Correlation Matrix of Normalized Biometric Variables in Male Hamadryas Baboons; Table S4: Individual Oxidative Stress Values and Comparison to Human Reference Ranges in Hamadryas Baboons.
