# Supplementary material for: Assessment of Oxidative Stress and Biometric Data in a Captive Colony of Hamadryas Baboons (Papio hamadryas Linnaeus, 1758) at the Ravenna Zoo Safari (Italy)
Source: Vet Sci. 2025 May 13;12(5):466. doi: 10.3390/vetsci12050466 (PMC12115884; doi:10.3390/vetsci12050466)
Supplement: Supplementary file 1 [file vetsci-12-00466-s001.zip › vetsci-3624976-supplementary.pdf]

Supplementary Materials

Table S1. Results of the normality test (Shapiro-Wilk test) performed on the biometric and oxidative stress data collected from males. For each variable, it was indicated whether the p-value was < 0.05 or statistically non-significant (ns); in other words, whether each variable followed a normal data distribution or not. BW = body weight; BL = body length; Oxy adsorbent test = measurements of the antioxidant barrier in serum samples; d-ROMs (U Carr) = determines the level of reactive oxygen metabolites expressed as Carratelli Units; OSi = Oxidative Stress Index.

Variable	*p*-value	Normal Distribution
**BW (Kg)**	**< 0.05**	No
**BL (mm)**	**< 0.05**	No
**Testis Volume (mm^3)**	ns	Yes
**Penile length (mm)**	**< 0.05**	No
***Baculum* length (mm)**	ns	Yes
**Oxy adsorbent test (mmol/ml)**	**< 0.05**	No
**d-ROMs (U Carr)**	ns	Yes
**OSi (d-ROMs/Oxy) %**	ns	Yes
**d-ROMs (mg H2O2/dL)**	ns	Yes

Table S2. Results of the normality test (Shapiro-Wilk test) performed on the biometric and oxidative stress data collected from females. For each variable, the *p*-value is reported as either < 0.05 or statistically not significant (ns), indicating whether the data distribution for each variable followed a normal distribution or not. BW = body weight; Oxy adsorbent test = measurements of the antioxidant barrier in serum samples; d-ROMs (U Carr) = determines the level of reactive oxygen metabolites expressed as Carratelli Units; OSi = Oxidative Stress index.

Variable	P-value	Normal Distribution
**BW (Kg)**	ns	Yes
**Oxy adsorbent test (mmol/ml)**	ns	Yes
**d-ROMs (U Carr)**	**< 0.05**	No
**OSi (d-ROMs/Oxy) %**	**< 0.05**	No
**d-ROMs (mg H2O2/dL)**	**< 0.05**	No

Table S3. Results of the Pearson correlation analysis performed on the normalized biometric data collected from males. For each variable, the correlation matrix values are reported below the diagonal, and the associated *p*-values are provided above the diagonal. BW = body weight; BL = body length; ns = not significative.

	**BW (Kg)**	**BL (mm)**	**Testis Volume (mm3)**	**Penile length (mm)**	***Baculum* length (mm)**
**BW (Kg)**		**< 0,05**	ns	**< 0,05**	**< 0,05**
**BL (mm)**	0.720		**< 0,05**	**< 0,05**	**< 0,05**
**Testis Volume (mm^3)**	0.225	0.499		**< 0,05**	ns
**Penile length (mm)**	0.864	0.819	0.323		**< 0,05**
***Baculum* length (mm)**	0.291	0.554	-0.104	0.302	

Table S4. Table with the oxidative stress values obtained for each individual, indicating whether each recorded value falls within the normal range (as considered for humans) or not. Oxy adsorbent test = measurements of the antioxidant barrier in serum samples; d-ROMs (U Carr) = determines the level of reactive oxygen metabolites expressed as Carratelli Units; OSi = Oxidative Stress index.

ID	Sex	Oxy adsorbent test (mmol/ml)		d-ROMs (U Carr)		OSI (d-Rom/Oxy) %		d-ROMs (mg H2O2/dL)		Overall_Status	Notes
1	Male	1275.7	Normal	163	Normal	12.8	Normal	13.04	Normal	Normal	
2	Male	1147.6	Normal	189	Normal	16.5	Normal	15.12	Normal	Normal	
3	Male	1409.2	Normal	208	Normal	14.8	Normal	16.64	Normal	Normal	
4	Male	1654.5	Normal	177	Normal	10.7	Normal	14.16	Normal	Normal	
5	Male	1279.2	Normal	196	Normal	15.3	Normal	15.68	Normal	Normal	
6	Male	1302.1	Normal	175	Normal	13.4	Normal	14	Normal	Normal	
7	Female	1308.5	Normal	218	Normal	16.7	Normal	17.44	Normal	Normal	
8	Male	1111.2	Normal	184	Normal	16.5	Normal	14.72	Normal	Normal	
9	Male	1338.5	Normal	233	Normal	17.4	Normal	18.64	Normal	Normal	
10	Male	1155.7	Normal	225	Normal	19.5	Normal	18	Normal	Normal	
11	Male	1155.3	Normal	211	Normal	18.3	Normal	16.88	Normal	Normal	
12	Male	1469.1	Normal	202	Normal	13.8	Normal	16.16	Normal	Normal	
13	Male	1226.9	Normal	238	Normal	19.4	Normal	19.04	Normal	Normal	
14	Female	1620.1	Normal	248	Normal	15.3	Normal	19.84	Normal	Normal	
15	Male	1194.2	Normal	198	Normal	16.6	Normal	15.84	Normal	Normal	
16	Male	1220.3	Normal	128	Normal	10.5	Normal	10.24	Normal	Normal	
17	Male	1235	Normal	175	Normal	14.2	Normal	14	Normal	Normal	
18	Female	1124.2	Normal	**666**	**Out of range**	59.2	Normal	**53.28**	**Out of range**	**Out of range**	**Pregnant**
19	Female	1437.8	Normal	**564**	**Out of range**	39.2	Normal	**45.12**	**Out of range**	**Out of range**	**Pregnant**
20	Male	1343.7	Normal	205	Normal	15.3	Normal	16.4	Normal	Normal	
21	Male	1530.8	Normal	226	Normal	14.8	Normal	18.08	Normal	Normal	
22	Male	1260.6	Normal	200	Normal	15.9	Normal	16	Normal	Normal	
23	Male	1150.4	Normal	185	Normal	16.1	Normal	14.8	Normal	Normal	
24	Male	1184.6	Normal	215	Normal	18.1	Normal	17.2	Normal	Normal	
25	Male	1137.1	Normal	252	Normal	22.2	Normal	20.16	Normal	Normal	
26	Male	1312.2	Normal	259	Normal	19.7	Normal	20.72	Normal	Normal	
27	Male	1297.1	Normal	274	Normal	21.1	Normal	21.92	Normal	Normal	
28	Male	1378.3	Normal	264	Normal	19.1	Normal	21.12	Normal	Normal	
29	Female	1301	Normal	**532**	**Out of range**	40.9	Normal	**42.56**	**Out of range**	**Out of range**	**Lactating**
30	Female	1788.5	Normal	196	Normal	11	Normal	15.68	Normal	Normal	
31	Female	1275.6	Normal	199	Normal	15.6	Normal	15.92	Normal	Normal	
32	Female	1380.8	Normal	197	Normal	14.3	Normal	15.76	Normal	Normal	
33	Female	1825.5	Normal	195	Normal	10.7	Normal	15.6	Normal	Normal	
34	Female	1330.8	Normal	230	Normal	17.3	Normal	18.4	Normal	Normal	
35	Male	1914.6	Normal	179	Normal	9.3	Normal	14.32	Normal	Normal	
36	Female	1385.2	Normal	149	Normal	10.7	Normal	11.92	Normal	Normal	
37	Male	1473.2	Normal	180	Normal	12.2	Normal	14.4	Normal	Normal	
38	Female	1421	Normal	221	Normal	15.5	Normal	17.68	Normal	Normal	
39	Female	1375.4	Normal	188	Normal	13.7	Normal	15.04	Normal	Normal	
41	Female	1618.9	Normal	292	Normal	18	Normal	23.36	Normal	Normal	
42	Male	1565.4	Normal	197	Normal	12.6	Normal	15.76	Normal	Normal	
43	Male	1966.3	Normal	166	Normal	8.4	Normal	13.28	Normal	Normal	

44	Female	1058.6	Normal	146	Normal	13.8	Normal	11.68	Normal	Normal	
45	Female	1071.3	Normal	131	Normal	12.2	Normal	10.48	Normal	Normal	
46	Female	1076.9	Normal	193	Normal	18	Normal	15.44	Normal	Normal	
47	Female	1618	Normal	72	Normal	4.5	Normal	5.76	Normal	Normal	
48	Female	1218.6	Normal	186	Normal	15.3	Normal	14.88	Normal	Normal	
49	Female	1181.7	Normal	202	Normal	17	Normal	16.16	Normal	Normal	
